# Whole-genome resequencing and genetic diversity of five indigenous cattle breeds from China

**DOI:** 10.1038/s41597-026-06610-y

**Published:** 2026-01-21

**Authors:** Wei Wang, Linxiang Li, Ying Chen, Xiaoqin Ma, Yueda Aguo, Jia Gan, Donghui Fang, Xiaodong Deng, Xiaoyun Chen, Fang He, Yi Shi, Changfeng Wu, Zhixin Yi, Yihui Chen, Maozhong Fu, Jun Yi

**Affiliations:** 1 Animal Genetic Breeding and Reproduction Key Laboratory of Sichuan Province, Sichuan Animal Science Academy, Chengdu, Sichuan 610066 China; 2 Bazhong Academy of Agriculture and Forestry Sciences, Bazhong, Sichuan 636000 China

**Keywords:** Structural variation, Animal breeding

## Abstract

China’s abundant indigenous yellow cattle resources are of great significance for studying environmental adaptability evolution, genetic resource conservation, and breeding improvement. The majority of the cattle population consists of indigenous breeds. Understanding the genetic architecture of these cattle breeds is essential for effective management and conservation efforts. In this study, we collected DNA samples from five local cattle breeds (n = 56) and obtained whole-genome sequencing (WGS) data for 10 Jinchuan (JC) yak samples from the NCBI database as the outgroup. Whole-genome resequencing generated approximately 2.3 TB of paired-end data, achieving an average depth of 13X and a depth range of 9.75X to 39.03X across the 66 samples. The sequencing data were pre-processed and mapped to the cattle reference genome (ARS-UCD1.2) with an alignment rate of 99.5%. Finally, the variant calling process produced approximately 31 million high-quality SNPs. These data enhance our understanding of cattle genetic architecture, enabling the discovery of functional variants and evolutionary insights to inform breeding strategies for climate-resilient and sustainable cattle production.

## Background & Summary

Indigenous animal genetic resources, particularly prominent in regions with rich agricultural traditions like China, hold vital reservoirs of global genetic diversity and are fundamental to the livelihoods of vast rural populations. Sichuan Province, in particular, serves as a significant hub for cattle diversity and breeding in China. Currently, the cattle population in Sichuan numbers in the millions, with a substantial proportion comprising diverse indigenous yellow cattle breeds. These local cattle breeds are typically named according to their distinctive coat colors, physical conformation, the ethnic communities raising them, and their specific geographic origins within the province. Through generations of adaptation, Sichuan yellow cattle have developed remarkable traits enabling them to thrive under challenging local conditions, including rugged mountainous terrain, variable seasonal climates (from humid summers to cool winters), seasonal limitations on high-quality forage, and endemic disease pressures^1–3^. These adaptive characteristics are the result of persistent natural selection within Sichuan’s distinct agroecological conditions, coupled with centuries of artificial selection by local farmers prioritizing resilience, draught power, and suitability to local farming systems.

To date, the Sichuan cattle breeding industry has been deeply integrated into the agro-pastoral composite production systems of the basin’s farming zones and hilly terrains, serving as a vital source of livelihood in the agro-pastoral regions of the western Sichuan Plateau. It has consistently served as a cornerstone of regional economic development. Despite their multifunctionality and pronounced phenotypic diversity, indigenous yellow cattle populations have suffered from inadequate systematic protection and conservation efforts. This deficiency has led to a significant loss of genetic resources and a noticeable decline in population size^4^. The ongoing crisis is primarily driven by complex factors, including unregulated hybridization during seasonal migration, intensified germplasm intermixing through socio-economic exchanges, and systemic challenges including: frequent flooding, disease outbreaks, uneven regional development and cross-province cattle movement^5^. These forces collectively accelerate the erosion of genetic diversity within local cattle populations. Therefore, a comprehensive understanding of Sichuan yellow cattle germplasm resources and genetic diversity is essential. Such foundational knowledge enables both improved management of livestock genetic resources and the design of scientifically informed breeding programs tailored to diverse production systems. In the context of national strategies aimed at developing a beef cattle industry belt in the southern mountainous regions and enhancing production systems in plateau pastoral areas, this research holds particular strategic and practical importance. Previous genome-wide studies have delineated a clear north-south genetic divergence and complex admixture history among Chinese indigenous cattle^6,7^. Furthermore, signatures of selection related to local adaptation have been identified in certain southern Chinese breeds^6^. However, a high-resolution, genome-wide characterization of the unique genetic architecture and adaptive evolution of Sichuan yellow cattle remains lacking.

For decades, quantitative genetic analysis has been viewed as a “black box” due to the inherently complex nature of gene action, which involves numerous loci of unknown effect and intricate interactions that collectively influence quantitative traits^8^. This complexity has impeded the elucidation of the underlying genetic mechanisms and the dissection of genetic architectures, thereby limiting the reproducibility of breeding outcomes across different spatial and temporal contexts. As such, there is a pressing need to unravel these intricate genetic processes with greater precision. Recent advances in genome sequencing, high-throughput SNP genotyping, and statistical genomics have catalyzed a shift in research focus from the analysis of neutral genetic variation to the identification and interpretation of functional variants^9^. In particular, the advent of whole-genome sequencing (WGS) has profoundly transformed our understanding of livestock genetics, enabling the detection of causal variants that are critical to improving animal production, health, and welfare, while also providing insights into the evolutionary history of domestic breeds^10,11^. Despite WGS becoming a standard methodology across numerous biological disciplines, including animal breeding, its routine application in the genetic characterization and evaluation of livestock genetic resources remains limited in many developing regions. In this study, we present WGS data from 56 Chinese Sichuan yellow cattle, sampled from a range of agro-ecological and climatic zones, including the Sichuan Basin and adjacent mountainous areas. This dataset constitutes a valuable genomic resource for advancing research on subtropical Indigenous cattle breeds. Our WGS data will enable a more comprehensive understanding of the distinct genetic makeup of Sichuan yellow cattle, facilitating the identification and functional validation of causal mutations associated with key economic traits such as heat and humidity tolerance, roughage utilization, and superior meat quality. Furthermore, these genomic insights will contribute to elucidating the adaptive and evolutionary dynamics of this important indigenous breed.

## Methods

### Sample collection, DNA extraction, and sequencing

**Fig. 1:**
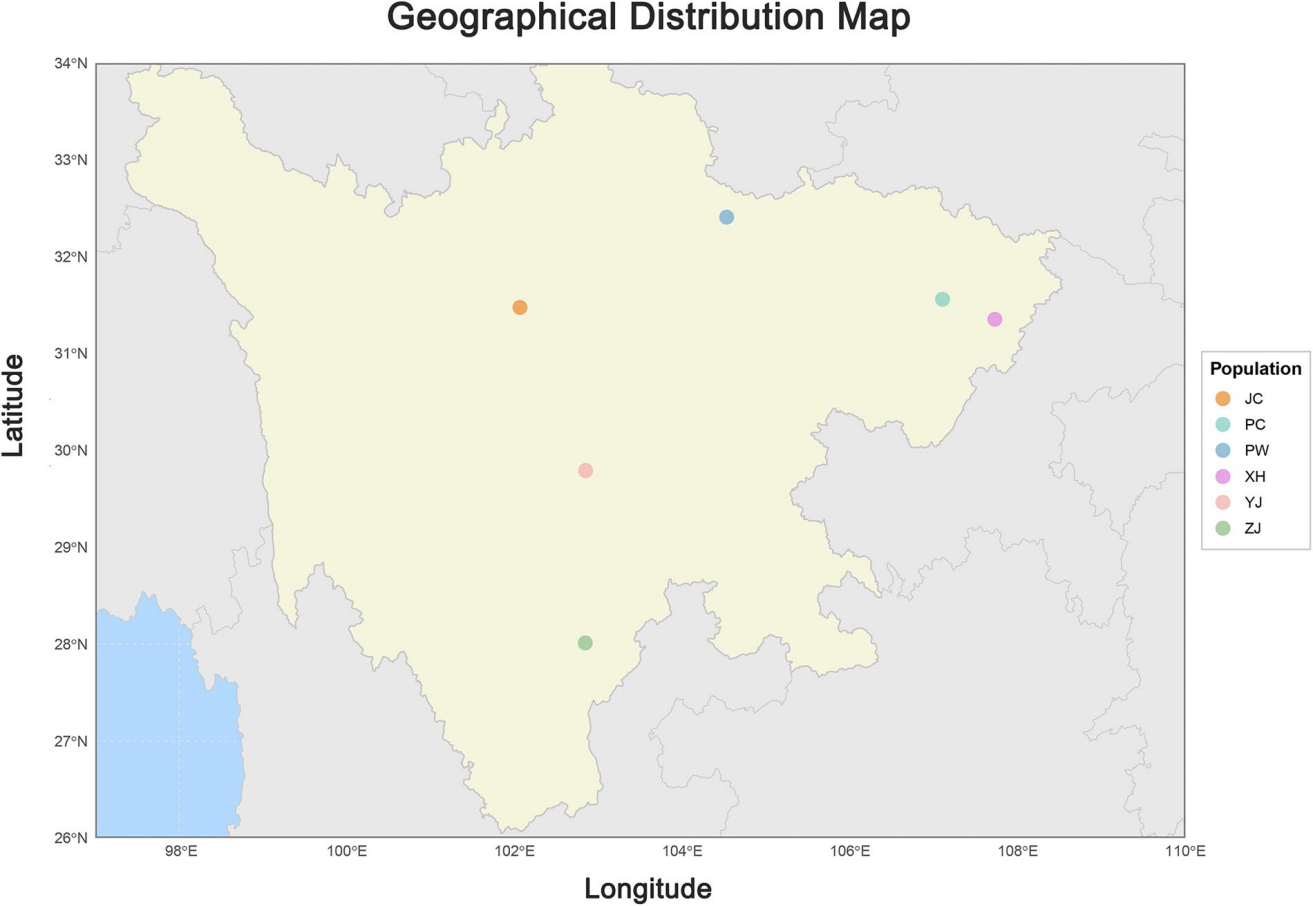
Geographical distribution of five indigenous yellow cattle breeds and one yak population in Sichuan Province, China. The map illustrates the sampling locations of all studied populations. PC represents the population in Pingchang County, Bazhong City, Sichuan Province, China; PW represents the population in Pingwu County, Mianyang City, Sichuan Province, China; YJ represents the population in Yingjing County, Ya’an City, Sichuan Province, China; XH represents the population in Xuanhan County, Dazhou City, Sichuan Province, China; ZJ represents the population in Zhaojue County, Liangshan Yi Autonomous Prefecture, Sichuan Province, China; JC represents the population in Jinchuan County, Aba Tibetan and Qiang Autonomous Prefecture, Sichuan Province, China.

For population genetic analysis in this study, a total of 56 blood samples were collected from indigenous cattle populations across five counties in Sichuan Province, China, including: Zhaojue (ZJ), Pingchang (PC), Xuanhan (XH), Pingwu (PW), and Yingjing (YJ) (Fig. 1). To ensure the reliability of genomic data, all sampled animals were confirmed to be healthy based on the following criteria: (1) clinical examination by a veterinarian to rule out obvious signs of disease; (2) a record of no major illness or medication for at least one months prior to sampling. All animal experimental procedures were approved by the Institutional Animal Care and Use Committee of Sichuan Academy of Animal Husbandry Science with approval number 2024023. The animals, all belonging to the indigenous yellow cattle species (*Bos taurus*), comprised five separate breeds. To provide an evolutionary reference and minimize analytical noise, we included ten JC yaks (*Bos grunniens*) as the outgroup in this study. These yaks were sampled from Jinchuan County, located in the Aba Tibetan and Qiang Autonomous Prefecture of Sichuan Province, China.

As a purebred high-altitude yak population with a low likelihood of hybridization, they provide a reliable genetic background for comparison. The sequencing data were downloaded from the NCBI database under BioProject accession number PRJNA483376^12^. All blood samples were obtained from healthy individuals under standard veterinary supervision, transported on the dry ice, and immediately stored at −80 °C to ensure DNA integrity. Detailed information on the sampling locations is provided in Supplementary Table 1. Genomic DNA was extracted from each blood sample using the Tianamp Genomic DNA Kit (TIANGEN, Beijing, China) according to the manufacturer’s instructions. The quality and concentration of extracted DNA were assessed using agarose gel electrophoresis and spectrophotometric analysis. Qualified DNA samples were used to construct sequencing libraries. These libraries were sequenced on the DNBSEQ-T7 platform to generate 150 bp paired-end reads (PE150), producing high-throughput whole-genome resequencing data for downstream population genomic analyses.

### Data preprocessing and read alignment

Raw sequencing data were quality-filtered with fastp (v0.23.2) using two criteria: (i) removal of reads containing >10% ambiguous (N) bases, and (ii) exclusion of reads with >20% of bases showing a Phred score < 5^13^. The resulting high-quality clean reads were then aligned to the Bos taurus reference genome (NCBI RefSeq assembly: GCF_002263795.3) using BWA-MEM2^14,15^. Following alignment, PCR duplicates in the BAM files were identified and removed using Samtools (v1.17)^16^.

### Detection of SNPs, InDels, SVs, and CNVs

SNP calling was conducted for each sample using the Genome Analysis Toolkit (GATK). Notably, the variant calling was performed jointly for all samples combined, rather than separately by population. This joint calling strategy was adopted to obtain a more comprehensive variant profile at the population level and to reduce false negatives that may arise from the limited sample size of individual subgroups. By integrating data across all populations, this approach improves the detection power for low-frequency variants and ensures consistency in variant discovery, thereby supporting the goal of constructing a high-quality genomic resource for future comparative studies.

In addition, InDels were detected using GATK following similar procedures. The structural variants (SVs) were identified using smoove (v0.2.8), a tool that integrates signals from both LUMPY and GRIDSS to enhance SV detection accuracy^17^. Copy number variations (CNVs) were detected using CNVnator (v0.8.2) with a bin size of 100 bp, a read-depth based approach developed by Alexander Handsaker and colleagues, which identifies both deletions and duplications across the genome^18^.

### Filtering of SNPs, InDels, SVs, and CNVs

The SNPs variants were filtered based on the following criteria: sequencing depth ≥ 4, missing rate < 0.1, and minor allele frequency (MAF) ≥ 0.05^19^. These thresholds were chosen to ensure reliable variant detection by balancing data quality with the retention of informative genetic variation. First, a minimum sequencing depth of ≥ 4 was applied to exclude low-confidence variants that may arise from random sequencing errors, while avoiding the loss of true polymorphisms in genomic regions with moderate coverage if a more stringent depth cutoff were used. Second, a missing rate < 0.1 ensured that each retained locus was genotyped in at least 90% of individuals, thereby reducing potential biases in downstream population genetic analyses such as principal component analysis (PCA) and population structure inference. Third, a minor allele frequency (MAF) ≥ 0.05 was used to remove extremely rare variants that are more likely attributable to sequencing artifacts and contribute little to population-level analyses, thus improving the robustness of the results. These thresholds are consistent with common practices in population genomics and were determined in consideration of the characteristics of our dataset and the objectives of this study.

InDel variants were filtered using thresholds commonly adopted in population genomics studies to ensure a balance between data quality and the retention of informative genetic diversity. The following criteria were applied: a minimum sequencing depth (DP) of 3 was applied to ensure sufficient read support for each variant site; a missing rate threshold of 0.3 was adopted, requiring that each variant be successfully genotyped in at least 70% of the samples to minimize potential bias in subsequent analyses; a minor allele frequency (MAF) cutoff of 0.05 was implemented to exclude extremely rare variants that are more likely to result from sequencing or alignment artifacts; and only biallelic sites were retained to simplify downstream analytical models genotyping ambiguity associated with multiallelic loci.

For SVs and CNVs, no additional quality-based filtering (e.g., on read depth or genotype missing rate) was applied, in contrast to the stringent thresholds used for SNPs and InDels. This decision was based on the consideration that SV and CNV callers (such as smoove and CNVnator) already integrate multiple internal quality metrics and evidence signals during the variant calling process, including read-pair, split-read, and depth-of-coverage information. Applying additional uniform quality thresholds—which are well-established for small variants but less standardized for large structural variants—could unnecessarily exclude valid SVs/CNVs due to the inherent differences in detection algorithms and the diverse nature of these variants. Therefore, to maintain consistency with common practices in structural variant analysis and to avoid the introduction of arbitrary biases, we relied on the built-in quality controls of the respective tools and performed filtering based primarily on biological relevance, retaining SVs between 50 bp and 1 Mb and CNVs between 1 kb and 1 Mb.

### Functional annotation of genetic variants

This study employed a Perl-based analytical pipeline to perform comprehensive functional annotation of genetic variants (SNPs and InDels) derived from 66 cattle samples. ANNOVAR (v2013-06-21) was used to annotate genomic variants, identifying their genomic positions and functional consequences^20^. The total genome size parameter was specified as 2,715,853,792 bp (including N bases). This integrated bioinformatics approach systematically combines reference genome sequences, structural gene annotations, and functional annotation databases to characterize bovine genomic variation at nucleotide resolution. The pipeline outputs detailed functional annotations for each genetic variant, enabling subsequent downstream analyses.

To systematically decipher the potential biological functions of SVs and CNVs, we performed genomic annotation for both types of variants using a standardized annotation pipeline. The workflow consisted of the following key steps: First, raw VCF files were preprocessed for format standardization using a custom Python script. Next, essential annotation information—including variant end position (END), number of supporting samples (SUPP), and variant length (SVLEN)—was extracted from the INFO field of the VCF files using an awk command, and subsequently converted into the eight-column input format required by the ANNOVAR tool. Using the ANNOVAR platform, comprehensive gene-region annotation was performed for all variant sites, covering gene bodies as well as their flanking 1000-bp regulatory regions. Finally, by integrating gene-region annotation results with coding-region functional annotations, we generated comprehensive functional annotation reports for both SVs and CNVs, which provided a foundation for downstream functional enrichment and biological pathway analyses. Data processing and visualization were carried out using custom scripts written in R, which were developed and provided by Beijing Bio Huaxing Gene Technology Co., LTD.

## Date Records

The dataset has been deposited in the NCBI Sequence Read Archive under accession number SRP655464^21^ and are also available through the China National GeneBank Database (CNGBdb; https://db.cngb.org/) under accession number CNP0007552^22^. All data are publicly accessible without restriction. In addition, the processed variant call datasets—including SNPs, SVs, CNVs, and indels—have been released via the Figshare repository (10.6084/m9.figshare.30759347)^23^. This comprehensive data deposition ensures full transparency and reproducibility, providing the global research community with unrestricted access to the complete set of genomic resources generated in this study.

## Technical Validation

### Evaluation of sequencing quality and performance

In this study, 56 samples were sequenced from five different populations of indigenous yellow cattle species in Sichuan, China for genetic diversity analysis. Additionally, we included 10 previously published JC yak samples from Sichuan as the outgroup. The sampling locations, including geographic coordinates and elevation information, were summarized in Supplementary Table 1. A total of 2,337 GB of raw data was generated, with an average Q20 value of 99.49% and a Q30 value reaching 96.68%. The quality control results indicated that the dataset had high sequencing quality; therefore, the BQSR step was not performed during variant calling. The GC content remained around 44.10% (see Supplementary Table 2). All samples were aligned to the ARS-UCD1.2 (GCF_002263795.3) reference genome, achieving an average alignment rate of 99.53%, with an average sequencing depth of 12.98X (Supplementary Table 3).

### Comprehensive SNP, InDel, SV, and CNV Profiling

**Fig. 2:**
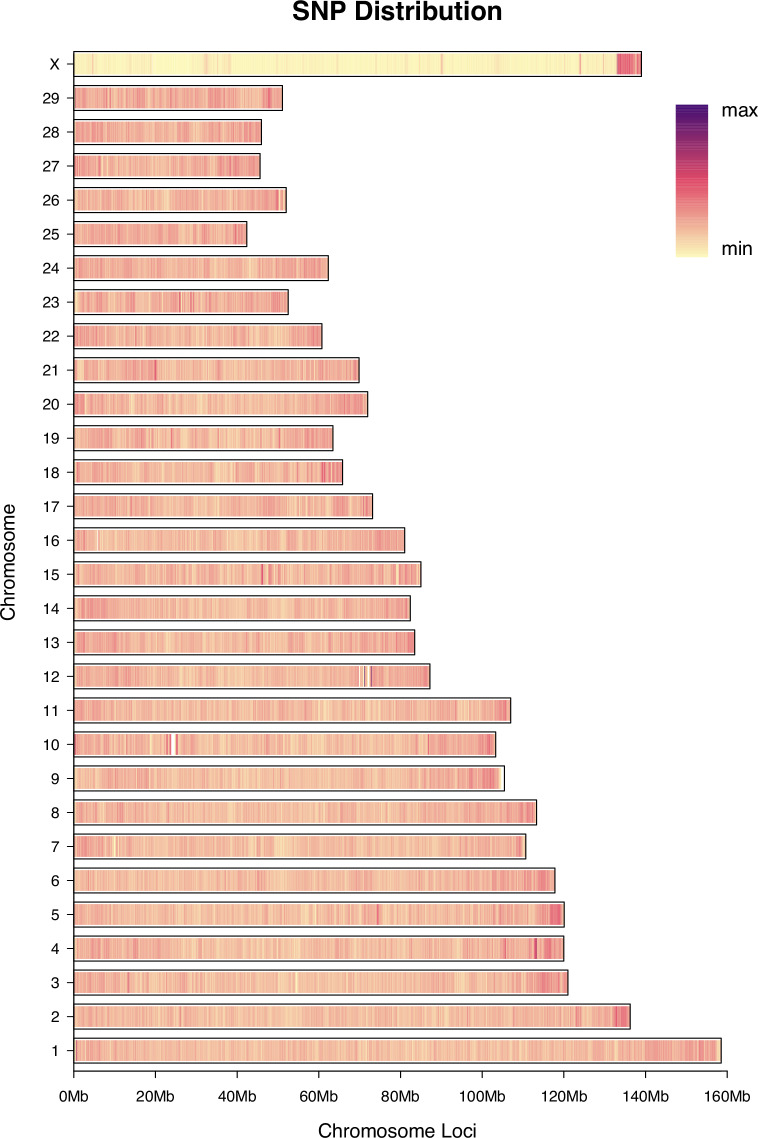
Genome-wide SNP density distribution across 66 samples. The length of each bar indicates the total variant count per chromosome, while the color gradient (light yellow to dark purple) shows the density distribution along the chromosome, from min to max. SNP, single nucleotide polymorphism.

Overall, the SNP density on autosomes (chromosomes 1–29) was generally higher than that on the X chromosome, which aligns with the expectation that sex chromosomes typically exhibit lower genetic diversity (Fig. 2). Subsequently, we performed functional annotation of all loci using ANNOVAR, and the results are summarized in Supplementary Table 4. The vast majority of SNPs were located in inter (18,335,329, 58.99%) and intronic regions (12,069,175, 38.83%), together accounting for 97.82% of the total SNPs. A total of 254,843 SNPs (0.82% of the total) were located in exonic regions, among which synonymous mutations (150,972, 0.49%) were more frequent than non-synonymous mutations (102,529, 0.33%). Additionally, we identified 1,170 stop-gain mutations and 172 stop-loss mutations, which may have significant impacts on protein function. In terms of mutation types, the numbers of transitions (ts) and transversions (tv) were 22,437,870 (72.19%) and 8,643,213 (27.81%), respectively, resulting in a ts/tv ratio of 2.60. This ratio is higher than the neutral evolution expectation (~2.0), suggesting the possible presence of purifying selection in the studied population or reflecting specific nucleotide composition and mutation biases in the bovine genome.

**Fig. 3:**
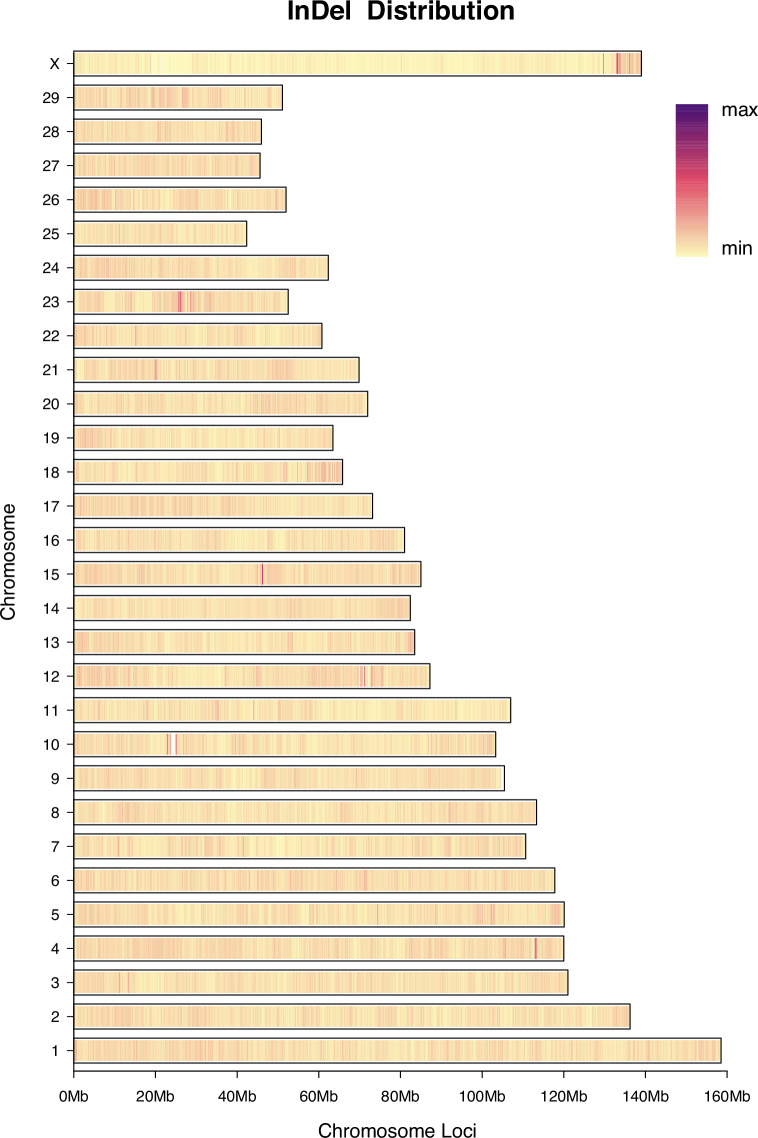
Genome-wide InDel density distribution across 66 samples. The length of each bar represents the total number of InDel variants per chromosome, and the color gradient reflects the density distribution along the chromosome, ranging from low (light yellow) to high (dark purple). InDel, insertion-deletion.

A comprehensive analysis of InDel variants identified a total of 2,337,297 high-quality InDels. Genomic functional annotation (Supplementary Table 5) revealed that the vast majority of InDels were located in intergenic (1,372, 58.72%) and intronic (898,773; 38.45%) regions, collectively accounting for 97.17% of all InDels. Regarding the distribution of InDel types, deletions (1,332,885; 57.03%) were more frequent than insertions (1,004,412; 42.97%), with an I/D ratio of approximately 0.75. Notably, at the chromosomal level, InDels on the X chromosome exhibited a density distribution pattern similar to that of SNPs, with high-density variant clusters observed in the telomeric regions (Fig. 3). However, compared to SNPs, the total number of InDels on the X chromosome, as well as across the entire genome, was significantly reduced, reflecting the generally lower mutation rate and stronger functional constraints characteristic of InDel variants.

**Fig. 4:**
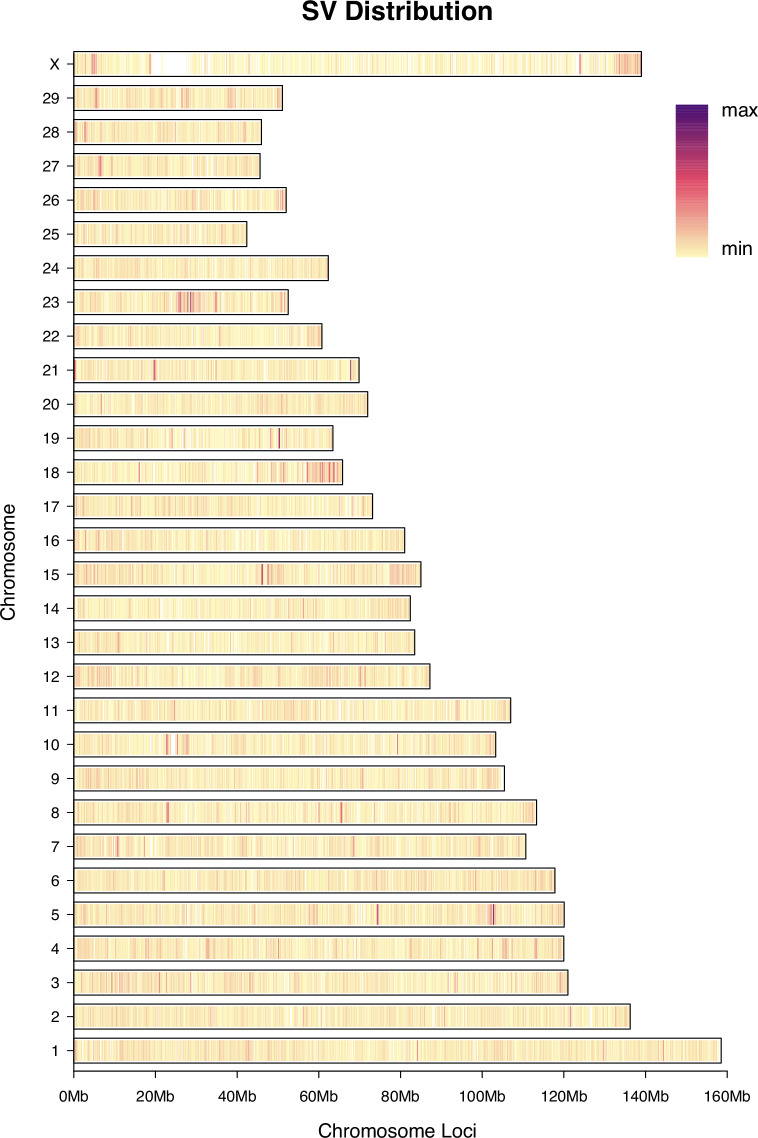
Genome-wide SV density distribution across 66 samples. The bar length indicates the total number of structural variants (SVs) detected on each chromosome, while the color gradient shows the distribution of SV density from low to high across genomic regions. SV, structural variation.

SVs were less abundant on the genome-wide scale compared to the other three variant types (SNP, InDel, and CNV). A total of 58,461 SVs were identified, with deletions (DEL) being the most predominant (50,070; 85.65%), followed by duplications (DUP, 5,079; 8.69%), and inversions (INV, 3,312; 5.67%) (Fig. 4; Supplementary Table 6). The majority of SVs were located in intergenic (32,234; 55.14%) and intronic (17,105; 29.26%) regions. Notably, similar to the pattern observed for SNPs, a high-density cluster of SVs was detected at the distal end of the X chromosome.

**Fig. 5:**
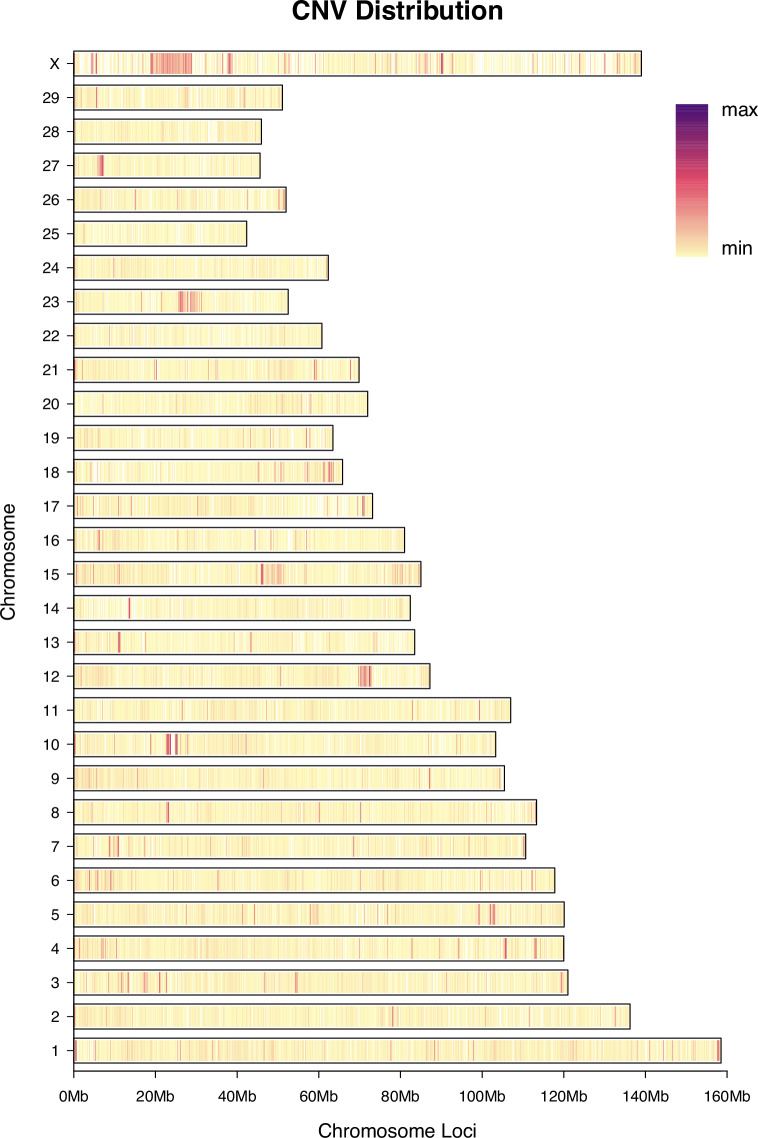
Genome-wide CNV density distribution across 66 samples. The length of each bar corresponds to the total number of copy number variation (CNV) events identified per chromosome, and the color gradient illustrates the density pattern of CNVs across the chromosome from low to high. CNV, copy number variation.

As illustrated in Fig. 5, copy number variations (CNVs) displayed a distinct pattern of genomic distribution characterized by pronounced regional clustering. Although the majority of CNV events were concentrated on the X chromosome, they did not show the marked enrichment at the distal telomeric ends that was observed for SNPs, InDels, and SVs. Notably, on the autosomes, the contrast between high- and low-density CNV regions was more striking, indicating a sharper demarcation of CNV-enriched and CNV-depleted segments compared to the relatively more uniform distributions of the other three variant types. In total, 242,516 high-confidence CNVs were identified, with deletions (DEL, 153,832; 63.43%) significantly outnumbering duplications (DUP, 88,684; 36.57%). Functional annotation revealed that the vast majority of CNVs were located in intergenic (155,467; 64.11%) and exonic (52,272; 21.55%) regions Supplementary Table 7. These findings suggest that CNVs are not only non-randomly distributed across the genome but also exhibit a chromosomal density profile that is distinct from that of SNPs, InDels, and SVs.

## Supplementary information

Supplementary tables

## Data Availability

The raw sequencing data generated in this study have been deposited in both the NCBI Sequence Read Archive under BioProject accession PRJNA1369724 (SRA: SRP655464)^21^ and the China National GeneBank DataBase (CNGBdb) under accession number CNP0007552^22^. The final variant sets (including SNPs, InDels, SVs, and CNVs) are available in the Figshare repository^23^.

## Code availability

Data analyses were primarily performed using standard bioinformatics tools within a Linux operating system environment. Detailed information regarding software versions and parameter settings is available at: https://github.com/triple-y/WGS-Chinese-yellow-cattle.
